# Carotid Plaque Features and Inflammatory Biomarkers as Predictors of Restenosis and Mortality Following Carotid Endarterectomy

**DOI:** 10.3390/ijerph192113934

**Published:** 2022-10-26

**Authors:** Raluca Niculescu, Eliza Russu, Emil Marian Arbănași, Réka Kaller, Eliza Mihaela Arbănași, Răzvan Marian Melinte, Cătălin Mircea Coșarcă, Iuliu Gabriel Cocuz, Adrian Horațiu Sabău, Andreea Cătălina Tinca, Adina Stoian, Vlad Vunvulea, Adrian Vasile Mureșan, Ovidiu Simion Cotoi

**Affiliations:** 1 Doctoral School of Medicine and Pharmacy, George Emil Palade University of Medicine, Pharmacy, Sciences and Technology of Targu Mures, 540142 Targu Mures, Romania; 2 Department of Pathology, Mures Clinical County Hospital, 540011 Targu Mures, Romania; 3 Clinic of Vascular Surgery, Mures County Emergency Hospital, 540136 Targu Mures, Romania; 4 Department of Surgery, George Emil Palade University of Medicine, Pharmacy, Science and Technology of Targu Mures, 540139, Targu Mures, Romania; 5 Faculty of Pharmacy, George Emil Palade University of Medicine, Pharmacy, Science and Technology of Targu Mures, 540139 Targu Mures, Romania; 6 Department of Orthopedics, Regina Maria Health Network, 540098 Targu Mures, Romania; 7 Department of Orthopedics, Humanitas MedLife Hospital, 400664 Cluj Napoca, Romania; 8 Department of Pathophysiology, George Emil Palade University of Medicine, Pharmacy, Science and Technology of Targu Mures, 540139 Targu Mures, Romania; 9 Department of Radiology, Mures County Emergency Hospital, 540136 Targu Mures, Romania

**Keywords:** MLR, NLR, PLR, SII, SIRI, AISI, carotid restenosis, carotid plaque, biomarkers

## Abstract

Background: Carotid endarterectomy (CEA) is the first-line surgical intervention for cases of severe carotid stenoses. Unfortunately, the restenosis rate is high after CEA. This study aims to demonstrate the predictive role of carotid plaque features and inflammatory biomarkers (monocyte-to-lymphocyte ratio (MLR), neutrophil-to-lymphocyte ratio (NLR), platelet-to-lymphocyte ratio (PLR), systemic inflammatory index (SII), Systemic Inflammation Response Index (SIRI), and Aggregate Index of Systemic Inflammation (AISI)) in carotid restenosis and mortality at 12 months following CEA. Methods: The present study was designed as an observational, analytical, retrospective cohort study and included all patients over 18 years of age with a minimum of 70% carotid stenosis and surgical indications for CEA admitted to the Vascular Surgery Clinic, Emergency County Hospital of Targu Mures, Romania between 2018 and 2021. Results: According to our results, the high pre-operative values of inflammatory biomarkers—MLR (OR: 10.37 and OR: 6.11; *p* < 0.001), NLR (OR: 34.22 and OR: 37.62; *p* < 0.001), PLR (OR: 12.02 and OR: 16.06; *p* < 0.001), SII (OR: 18.11 and OR: 31.70; *p* < 0.001), SIRI (OR: 16.64 and OR: 9.89; *p* < 0.001), and AISI (OR: 16.80 and OR: 8.24; *p* < 0.001)—are strong independent factors predicting the risk of 12-month restenosis and mortality following CEA. Moreover, unstable plaque (OR: 2.83, *p* < 0.001 and OR: 2.40, *p* = 0.04) and MI (OR: 3.16, *p* < 0.001 and OR: 2.83, *p* = 0.005) were independent predictors of all outcomes. Furthermore, AH (OR: 2.30; *p* = 0.006), AF (OR: 1.74; *p* = 0.02), tobacco (OR: 2.25; *p* < 0.001), obesity (OR: 1.90; *p* = 0.02), and thrombotic plaques (OR: 2.77; *p* < 0.001) were all independent predictors of restenosis, but not for mortality in all patients. In contrast, antiplatelet (OR: 0.46; *p* = 0.004), statin (OR: 0.59; *p* = 0.04), and ezetimibe (OR:0.45; *p* = 0.03) therapy were protective factors against restenosis, but not for mortality. Conclusions: Our data revealed that higher preoperative inflammatory biomarker values highly predict 12-month restenosis and mortality following CEA. Furthermore, age above 70, unstable plaque, cardiovascular disease, and dyslipidemia were risk factors for all outcomes. Additionally, AH, AF, smoking, and obesity were all independent predictors of restenosis but not of mortality in all patients. Antiplatelet and statin medication, on the other hand, were protective against restenosis but not against mortality.

## 1. Introduction

Stroke is one of the medical emergencies that presents a high mortality rate, currently occupying second place worldwide as a cause of mortality [1,2,3]. The main mechanism underlying ischemic stroke is atherosclerosis, which is represented by the formation and progression of atherosclerotic plaques in the carotid arteries [4,5,6]. Often, atherosclerotic plaque does not generate specific symptoms, but vulnerable/unstable plaques present an increased risk of ischemic stroke [4,5].

Carotid endarterectomy (CEA) is the most effective, first-line surgical intervention for cases of severe carotid stenoses [7,8,9]. CEA is performed by surgical removal of atherosclerotic plaque from the level of the bifurcation of the common carotid artery and the level of the internal carotid artery, to reduce the risk of developing a stroke [10]. According to published studies, the rate of post-endarterectomy restenosis ranges from 5 to 37% depending on the definition of restenosis and the follow-up duration [11,12,13].

The presence of risk factors (smoking, obesity, dyslipidemia), as well as the local and systemic inflammatory response are involved in post-endarterectomy restenosis [14,15,16]. Regarding the characteristics of atherosclerotic plaques, these were associated with the negative evolution of the patient with ischemic stroke [17,18,19,20,21], as well as with stent restenosis [22].

Among the systemic inflammatory biomarkers, the most accessible and easy to implement in current medical practice are hematological reports based on the total number of monocytes, neutrophils, lymphocytes, and platelets, respectively: monocyte-to-lymphocyte ratio (MLR), neutrophil-to-lymphocyte ratio (NLR), platelet-to-lymphocyte ratio (PLR), systemic inflammatory index (SII), systemic Inflammation Response Index (SIRI), and aggregate index of systemic inflammation (AISI), whose predictive role has been demonstrated in the case of cardiovascular pathologies [17,18,19,20,21,22,23], oncological pathologies [24,25,26,27], chronic kidney disease [28,29,30], and, more recently, in the case of COVID-19 patients [31,32,33,34,35,36,37].

This study aims to demonstrate the predictive role of carotid plaque features and systemic inflammatory biomarkers in carotid restenosis and mortality at 12 months following CEA.

## 2. Materials and Methods

### 2.1. Study Design

The present study was designed as an observational, analytical, retrospective cohort study and included all patients over 18 years of age with a minimum of 70% carotid stenosis and surgical indications for CEA admitted to the Vascular Surgery Clinic, Emergency County Hospital of Targu Mures, Romania between 2018 and 2021. Patients with active tumors, hematological disease, restenosis after CEA, or contralateral CEA were all excluded. Regarding restenosis at 12 months, all patients enrolled in this study were initially divided into two groups named “No Stenosis” and “Restenosis”. The ideal cut-off value for all inflammatory biomarkers was used to calculate 12-month restenosis and mortality.

### 2.2. Data Collection

The patient’s age, gender, and hospitalization period were extracted from the hospital’s electronic database. Regarding comorbidities, the following cardiac pathologies were recorded: arterial hypertension (AH), atrial fibrillation (AF), ischemic heart disease (IHD), history of myocardial infarction (MI), chronic heart failure (CHF), as well as other pathologies: chronic kidney disease (CKD), peripheral arterial disease (PAD), and diabetes mellitus (DM).

The following were extracted from the pre-operative laboratory analyses: hemoglobin level, hematocrit level, number of neutrophils, monocytes, platelets, lymphocytes, glucose level, total cholesterol level, triglyceride level, blood urea nitrogen (BUN), creatinine, and Glomerular filtration rate (GFR).

### 2.3. Inflammatory Markers

Inflammatory biomarkers were determined from the first blood test result. The ratio was calculated using the equations:

- MLR = monocytes/lymphocytes
- NLR = neutrophils/lymphocytes
- PLR = platelets/lymphocytes
- SII = (neutrophils * platelets)/lymphocytes
- SIRI = (monocytes * platelets)/lymphocytes
- AISI = (neutrophils * monocytes * platelets)/lymphocytes

### 2.4. Surgical Technique

All patients were operated on by the same surgical team, under cervical block. The surgical intervention was carried out in the conventional method, the common, internal, and external carotid artery being prepared first, followed by clamping at the level of the three carotid arteries. Before clamping, 5000 IU of intravenous heparin was administered, and in case of post-clamping neurological changes, an intravascular shunt (FlexcelTM Carotid Shunt, LeMaitre^®^, North Brunswick, NJ, USA) was used. Later, carotid endarterectomy was performed through longitudinal arteriotomy, with removal of the atherosclerotic plaque, and sent for histological evaluation. Finally, carotid artery reconstruction was performed using a 6:0 prolene thread and an intradermal suture (Ethicon, Norderstedt, Germany).

### 2.5. Study Outcomes

The primary endpoints were the occurrence of restenosis higher than 50% and mortality at 12 months. The number of days spent in the hospital was recorded as a secondary outcome. The primary outcomes were stratified for the optimal cut-off value of inflammatory biomarkers.

### 2.6. Follow-Up Strategy

Patients were evaluated by Doppler ultrasound, 4 weeks, 6 months, and 12 months after the intervention. Restenosis was recorded if, during the ultrasonographic examination, at the level of the internal carotid artery, a stenosis of at least 50% was detected after CEA. If patients did not show up for their control visits and did not phone ahead of time to reschedule, the family was called to find out the patient’s situation. Mortality was recorded through telephone contact with the family.

### 2.7. Histopathological and Morphometrical Analysis

The samples were taken from the intern carotid artery. The carotid plaque was immersed in formalin 4% in a container that was at least five times the size of the fragment (at room temperature). The routine Hematoxylin–Eosin staining protocol was followed (the pieces were fixed, placed in 70% alcohol for one hour, 96% alcohol for a maximum of 24 h, then absolute alcohol for one hour, and then two xylene baths were performed, after which the tissue was placed in two paraffin baths of 30 min each, and it was sectioned at the microtomy in three sections at different levels with dimensions of 3 microns, and then the staining process followed). The procedure for Red Oil staining was used to examine the fatty deposits. There was no decalcification for any type of lesion. The histological type of the atherosclerotic plaques was analyzed and classified using the classification proposed by Virmani et al. [38], with consideration of morphopathological features such as intimal thickness, the presence of calcifications, fatty deposits, and plaque rupture.

### 2.8. Statistical Analysis

SPSS for Mac OS version 28.0.1.0 was used for statistical analysis (SPSS, Inc., Chicago, IL, USA). Chi-square tests were used to assess the associations of the ratios with category factors, while Student’s *t*- or Mann–Whitney tests were used to assess differences in continuous variables. To analyze the predictive power and to establish the cut-off values of inflammatory biomarkers, receiver operating characteristic (ROC) curve analysis was utilized. The ROC curve analysis was used to determine the appropriate MLR, NLR, PLR, SII, SIRI, and AISI cut-off values based on the Youden index (Youden Index = Sensitivity + Specificity − 1, ranging from 0 to 1). To identify independent predictors of 12-month restenosis and mortality after CEA, a multivariate logistic regression analysis using variables with *p* < 0.1 was undertaken.

## 3. Results

During the studied period, 369 patients underwent carotid endarterectomy. Of the patients, 190 were male (51.49%), and the mean age was 71.33 ± 11.61 (39–94). At the pre-operative carotid ultrasound, at the level of the symptomatic carotid, 268 patients presented stenoses between 70 and 90%, while 101 patients had stenoses greater than 90% but with no occlusion. At the histopathological analysis of the extracted plaques, 213 (57.72%) were stable plaques, and 156 (42.28%) showed signs of instability. Pre-operative pharmacological therapy included anticoagulant medication for 95 patients (25.75%), antiplatelet medication for 178 patients (48.24%), and statins for 222 patients (60.16%). Over the 12 months, 38 patients died (10.38%). The rest of the recorded variables are presented in Table 1.

Patients whose restenosis occurred in the 12 months following CEA were older (*p* < 0.0001), had higher incidences of cardio-vascular comorbidities (AH (*p* = 0.006), IHD (*p* = 0.001), AF (*p* < 0.0001), and MI (*p* < 0.0001)), and higher incidences of all risk factors enrolled, as seen in Table 1. In terms of ICA stenosis and carotid plaque features, in the restenosis group was a higher incidence of severe ipsilateral stenosis (90–99%; *p* < 0.0001), as well a higher incidence of unstable plaques (*p* = 0.0001) and especially thrombotic plaque (*p* = 0.0004). As pre-operative drug therapy, patients with no stenosis for 12-month follow-up had a higher incidence of antiplatelet use (*p* = 0.004) and ezetimibe (*p* = 0.03).

Regarding the laboratory findings, patients in the restenosis group had higher bun (*p* < 0.0001), creatinine (*p* = 0.0003), neutrophil (*p* < 0.0001), monocyte (*p* = 0.003), platelet (*p* = 0.03), and all systemic inflammatory biomarker (*p* < 0.0001) values as well as lower hemoglobin (*p* = 0.0004), hematocrit (*p* = 0.003), GFR (*p* < 0.0001), and lymphocyte (*p* < 0.0001). Moreover, there were higher incidences of mortality (*p* < 0.0001) Table 1.

**Figure 1:**
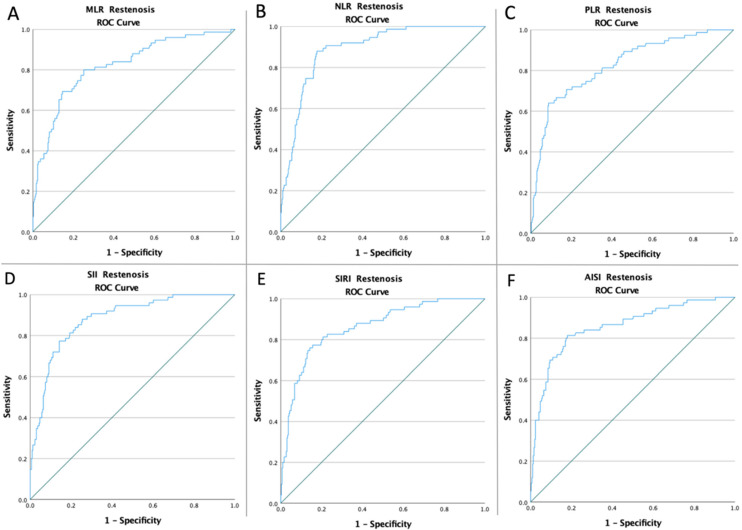
ROC curve analysis concerning Restenosis (**A**) for the MLR (AUC: 0.822; *p* < 0.0001), (**B**) for the NLR (AUC: 0.890; *p* < 0.0001), (**C**) for the PLR (AUC: 0.825; *p* < 0.0001), (**D**) for the SII (AUC: 0.880; *p* < 0.0001), (**E**) for the SIRI (AUC: 0.861; *p* < 0.0001), and (**F**) for the AISI (AUC: 0.857; *p* < 0.0001).

**Figure 2:**
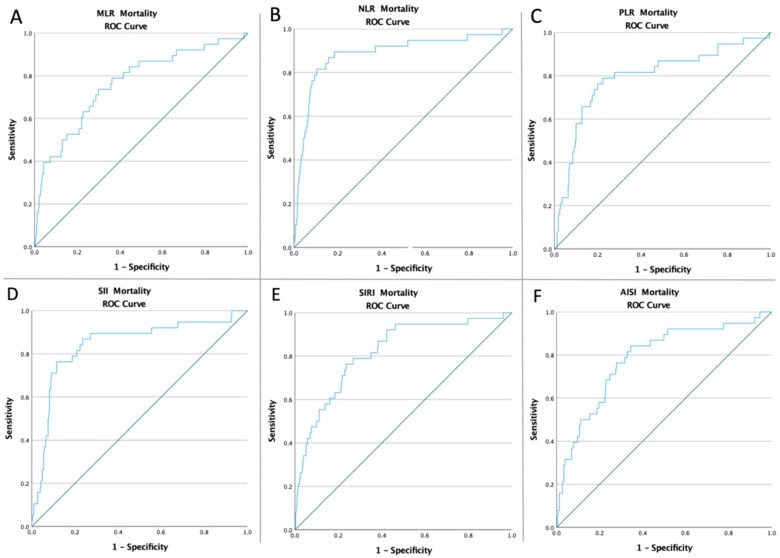
ROC curve analysis concerning Mortality (**A**) for the MLR (AUC: 0.765; *p* < 0.0001), (**B**) for the NLR (AUC: 0.885; *p* < 0.0001), (**C**) for the PLR (AUC: 0.794; *p* < 0.0001), (**D**) for the SII (AUC: 0.844; *p* < 0.0001), (**E**) for the SIRI (AUC: 0.819; *p* < 0.0001), and (**F**) for the AISI (AUC: 0.784; *p* < 0.0001).

The ROC curves of all inflammatory biomarkers were created to determine whether the baseline of these markers was predictive of 12-month restenosis and mortality following CEA (Figure 1 and Figure 2). The optimal cut-off value obtained from Youden’s index, areas under the curve (AUC), and the predictive accuracy of the markers are listed in Table 2.

The restenosis risk, mortality, and length of hospital stay were further analyzed after dividing the patients into paired groups according to the optimal cut-off value of inflammatory biomarkers. Moreover, there was a higher incidence of restenosis risk and mortality rate for all the inflammatory biomarkers, as seen in Table 3.

A multivariate analysis was used to determine the association between the inflammatory biomarkers, underlying risk factors, restenosis, and mortality risk at 12 months following CEA. A high baseline value of all systemic inflammatory markers was a strong independent predictor of restenosis and mortality (for all *p* < 0.0001). Moreover, as shown in Table 4, age above 70 (OR: 3.26; *p* < 0.001 and OR: 5.44; *p* = 0.007), MI (OR: 3.16; *p* < 0.001 and OR: 2.83; *p* = 0.005), dyslipidemia (OR: 5.27; *p* < 0.001 and OR: 2.80; *p* = 0.005), and unstable plaques (OR: 2.83; *p* < 0.001 and OR: 2.40; *p* = 0.04) were all independent predictors of restenosis and mortality. Furthermore, AH (OR: 2.30; *p* = 0.006), AF (OR: 1.74; *p* = 0.02), tobacco (OR: 2.25; *p* < 0.001), obesity (OR: 1.90; *p* = 0.02), and thrombotic plaques (OR: 2.77; *p* < 0.001) were all independent predictors of restenosis, but not for mortality in all patients. In contrast, antiplatelet (OR: 0.46; *p* = 0.004), statin (OR: 0.59; *p* = 0.04), and ezetimibe (OR:0.45; *p* = 0.03) therapy were protective factors against restenosis, but not for mortality (Table 4).

## 4. Discussion

The role of inflammation in the progression of atherosclerosis is well-acknowledged [39,40,41]. Gibson et al. [42], Tamhane et al. [43], and Duffy et al. [44] demonstrated the role of neutrophils and lymphocytes in the negative evolution of patients with coronary disease through the progression of coronary atherosclerotic plaques. Furthermore, the applicability of some of the inflammatory biomarkers analyzed in this research (NLR, PLR, and MLR) has been demonstrated in the negative evolution of patients with acute ischemic stroke [45,46,47,48,49], as well as restenosis and neurological complications following CEA and carotid stenting [50,51,52,53,54,55].

According to our results, the high pre-operative values of inflammatory biomarkers—MLR (OR: 10.37 and OR: 6.11; *p* < 0.001), NLR (OR: 34.22 and OR: 37.62; *p* < 0.001), PLR (OR: 12.02 and OR: 16.06; *p* < 0.001), SII (OR: 18.11 and OR: 31.70; *p* < 0.001), SIRI (OR: 16.64 and OR: 9.89; *p* < 0.001), and AISI (OR: 16.80 and OR: 8.24; *p* < 0.001)—are strong independent factors predicting the risk of 12-month restenosis and mortality following CEA. Moreover, unstable plaque (OR: 2.83, *p* < 0.001 and OR: 2.40, *p* = 0.04) and MI (OR: 3.16, *p* < 0.001 and OR: 2.83, *p* = 0.005) were independent predictors of all outcomes, as seen in Table 4.

Similar to our findings, older patients, cardiovascular diseases, smoking, obesity, and dyslipidemia have all been identified as risk factors for restenosis following CEA [16,56,57]. Furthermore, Zhou et al. [58] and Hellings et al. [59] demonstrated that the histopathological characteristics of carotid plaques are associated with carotid restenosis after CEA. 

In studies recently reported in the literature, the NLR and PLR have been assessed regarding restenosis and poor outcome following ECA. Thus, Halazun et al. [51] found that high values of NLR > 5 (OR, 3.38; 95% CI, 1.81–6.27; *p* < 0.001) were associated with cognitive dysfunction in 432 CEA patients. Furthermore, King et al. [52] reported a correlation between pre-operative values of NLR > 3 and an increased risk of stroke and death following CEA for asymptomatic carotid artery stenosis. According to Deşer et al. [53], PLR > 145,304 (83.3% sensitivity, 73.8% specificity; *p* = 0.002) is an independent predictor for stroke (*p* = 0.047).

In the case of 285 patients with acute ischemic stroke, Sadeghi et al. found that high values of NLR > 5.73, 24 h after thrombolysis, are associated with a poor functional prognosis [45]. Similarly, Lee et al. recently published research in which they proved that high NLR > 6.2 and PLR > 103.6 levels are predictive factors for failed reperfusion following endovascular treatment in 282 patients with acute ischemic stroke [46]. Furthermore, Lattanzi et al. demonstrated, in two articles, that NLR > 6.4 (OR: 1.11; 95% CI: 1.04–1.18; *p* = 0.001) predicts early neurological deterioration [47,52] and SIRI > 3.8 was an independent predictor of futile recanalization (OR: 2.88; 95% CI: 1.56–5.30; *p* < 0.001) [60,61] for ischemic stroke patients undergoing endovascular treatment.

Scicchitano et al. [61] and Marzullo et al. [62] studied and established that the soluble suppressor of tumorigenicity (sST-2) works as an independent predictor of mortality and carotid plaque in individuals with carotid atherosclerotic plaque following CEA. The relevance of nutraceutical and dietary carotenoid supplementation in the daily diet in terms of dyslipidemia and lipidic management was established by two systematic reviews published by Scicchitano et al. [63] and Ciccone et al. [64].

The primary outcome of this research is that pre-operative systemic inflammatory biomarkers have a high predictive role in the risk of restenosis occurrence and mortality following CEA. As seen in Table 4, cardiovascular diseases (AH, AF, and MI), unstable plaques, elderly patients, and other risk factors (tobacco, obesity, and dyslipidemia) all predict 12-month restenosis. Moreover, antiplatelet, statin, and ezetimibe medication are protective factors for 12-month restenosis after CEA. To the best of our knowledge, this is the first research to evaluate the predictive role of carotid plaque morphology, pre-operative drug therapy, red blood cell biomarkers, and risk of restenosis and mortality at 12 months following CEA.

Although this study included 369 patients who underwent CEA for four years and had significant results with the high level of sensitivity and specificity of the analyzed inflammatory biomarkers in the prediction of restenosis and mortality at 12 months after CEA, it has certain limitations.

### Limitations

Our study has some limitations. Firstly, we must consider the monocentric and retrospective design of the study. Secondly, we excluded patients who benefited from carotid stenting, or patients with contralateral CEA. Moreover, regarding the exclusion criteria (malignancy and hematological disease), the results cannot be extrapolated to the general population. In the future, we recommend a multicentric prospective study, following the inflammatory status and poor outcomes of patients after CEA. Furthermore, additional research is necessary to support our findings.

## 5. Conclusions

Our data revealed that higher preoperative inflammatory biomarker values (MLR, NLR, PLR, SII, SIRI, and AISI) highly predict 12-month restenosis and mortality following CEA. Furthermore, during the study period, age above 70, unstable plaque, cardiovascular disease, and dyslipidemia were risk factors for 12-month restenosis and mortality. Additionally, AH, AF, smoking, and obesity were all independent predictors of restenosis but not of mortality in all patients. Antiplatelet, statin, and ezetimibe medication, on the other hand, were protective against restenosis but not against mortality. Given their ease of use and inexpensive cost, these ratios can be used for preoperative-risk group stratification and improved patient management of restenosis following CEA.

## Figures and Tables

**Table 1:** The baseline characteristic data of all patients and divided according to the restenosis risk at 24 months.

Variables	All Patients n = 369	No Stenosis n = 294	Restenosis n = 75	*p*-Value (OR; CI 95%)
Age mean ± SD (min–max)	71.33 ± 11.61 (39–94)	69.91 ± 11.35 (39–91)	76.90 ± 10.97 (44–94)	<0.0001
Male/Female sex no. (%)	190 (51.49%) 179 (48.51%)	157 (53.30%) 137 (46.60%)	33 (44.00%) 42 (56.00%)	0.14 (1.45; 0.87–2.42)
**Comorbidities and Risk factors**				
AH, no. (%)	240 (65.04%)	181 (61.56%)	59 (78.67%)	0.006 (2.30; 1.26–4.19)
IHD, no. (%)	146 (39.57%)	104 (35.37%)	42 (56.00%)	0.001 (2.32; 1.38–3.89)
AF, no. (%)	105 (28.46%)	68 (23.13%)	37 (49.33%)	<0.0001 (3.23; 1.90–5.48)
CHF, no. (%)	84 (22.76%)	66 (22.45%)	18 (24.00%)	0.77 (1.09; 0.60–1.98)
MI, no. (%)	88 (23.85%)	56 (19.05%)	32 (42.67%)	<0.0001 (3.16; 1.83–5.44)
DM, no. (%)	108 (29.27%)	82 (27.89%)	26 (34.67%)	0.25 (1.37; 0.79–2.35)
CKD, no. (%)	69 (18.70%)	54 (18.37%)	15 (20.00%)	0.74 (1.11; 0.58–2.10)
PAD, no. (%)	86 (23.31%)	67 (22.79%)	19 (25.33%)	0.64 (1.14; 0.63–2.06)
Tobacco, no. (%)	80 (21.68%)	53 (18.03%)	27 (36.00%)	0.001 (2.55; 1.46–4.46)
Obesity, no. (%)	94 (25.47%)	67 (22.79%)	27 (36.00%)	0.02 (1.90; 1.10–3.28)
Dyslipidemia, no. (%)	114 (30.89%)	68 (23.13%)	46 (61.33%)	<0.0001 (5.27; 3.07–9.02)
**Ipsilateral ICA Stenosis**				
70–90%, no. (%)	268 (72.62%)	242 (82.31%)	26 (34.67%)	<0.0001 (8.77; 4.99–15.38)
90–99%, no. (%)	101 (27.37%)	52 (17.68%)	49 (65.33%)	
**Contralateral ICA Stenosis**				
<50%, no. (%)	218 (59.07%)	176 (59.86%)	42 (56%)	0.54 (0.85; 0.51–1.42)
50–70%, no. (%)	89 (24.11%)	69 (23.46%)	20 (26.67%)	0.56 (1.18; 0.66–2.11)
>70%, no. (%)	62 (16.80%)	49 (16.67%)	13 (17.33%)	0.89 (1.04; 0.53–2.05)
** Histological Type of Carotid Plaque **				
** Stable Plaques, ** **no. (%)**	213 (57.72%)	169 (57.48%)	44 (58.66%)	0.85 (1.04; 0.62–1.75)
Fibroatheroma, no. (%)	88 (23.85%)	65 (22.10%)	23 (30.67%)	0.12 (1.55; 0.88–2.73)
Fibrocalcific, no. (%)	125 (33.88%)	104 (35.37%)	21 (28%)	0.22 (0.71; 0.40–1.24)
** Unstable Plaques, ** **no. (%)**	156 (42.28%)	109 (37.07%)	47 (62.67%)	0.0001 (2.84; 1.68–4.81)
Thrombotic Plaque, no. (%)	73 (19.78%)	47 (15.98%)	26 (34.67%)	0.0004 (2.78; 1.57–4.92)
With A Thrombus in Organization, no. (%)	41 (11.11%)	30 (10.20%)	11 (14.67%)	0.27 (1.51; 0.71–3.17)
Thin-Cap Fibro-Atheroma, no. (%)	26 (7.05%)	20 (6.80%)	6 (8%)	0.71 (1.19; 0.46–3.07)
Calcified Nodule, no. (%)	16 (4.34%)	12 (4.08%)	4 (5.33%)	0.63 (1.32; 0.41–4.22)
**Pre-Operative Drug Therapy**				
Anticoagulant, no. (%)	95 (25.75%)	78 (26.53%)	17 (22.67%)	0.49 (0.81; 0.44–1.47)
Antiplatelet, no. (%)	178 (48.24%)	153 (52.04%)	25 (33.33%)	0.004 (0.46; 0.27–0.78)
Statins, no. (%)	222 (60.16%)	184 (62.59%)	38 (50.67%)	0.06 (0.61; 0.36–1.02)
Ezetimibe, no. (%)	77 (20.86%)	68 (23.12%)	9 (12%)	0.03 (0.45; 0.21–0.95)
PCSK9I, no. (%)	21 (5.69%)	18 (6.12%)	3 (4%)	0.48 (0.63; 0.18–2.22)
**Laboratory data**				
Hemoglobin g/dL median (Q1–Q3)	13.7 (12.5–14.86)	13.81 (12.75–14.99)	13.2 (10.96–14.4)	0.0004
Hematocrit % median (Q1–Q3)	41.9 (38.2–45)	42.0 (39.02–45.01)	41.23 (34.27–44.5)	0.003
Glucose mg/dL median (Q1–Q3)	115 (95–145.8)	110 (94–133.7)	146 (120.75–173.75)	<0.0001
Cholesterol mg/dL median (Q1–Q3)	178.2 (146.4–215.2)	177.75 (148.02–214.07)	180.1 (146–239.25)	0.09
Triglyceride mg/dL median (Q1–Q3)	119.1 (90.7–165.3)	117.35 (93.55–163.67)	123.1 (81.4–170.15)	0.22
GFR (mL/min/1.73 m^2^) median (Q1–Q3)	76.06 (57.47–92.24)	77.93 (63.28–93.46)	62.44 (37.42–86.08)	<0.0001
BUN mg/dL median (Q1–Q3)	41.1 (31.2–54.8)	39.15 (30.05–50.6)	51.2 (37.75–97.2)	<0.0001
Creatinine mg/dL median (Q1–Q3)	0.90 (0.76–1.12)	0.89 (0.75–1.08)	1.10 (0.78–1.52)	0.0003
Neutrophils ×10³/µL median (Q1–Q3)	4.78 (3.47–7.12)	4.33 (3.33–6.04)	7.79 (5.43–9.85)	<0.0001
Lymphocytes ×10³/µL median (Q1–Q3)	2.10 (1.48–2.87)	2.35 (1.70–3.19)	1.36 (0.79–1.72)	<0.0001
Monocyte ×10³/µL median (Q1–Q3)	0.53 (0.4–0.72)	0.51 (0.4–0.68)	0.60 (0.4–0.92)	0.003
PLT ×10³/µL median (Q1–Q3)	234 (189.4–280)	234 (188.62–277.57)	240.9 (199–323.85)	0.03
MLR, median (Q1–Q3)	0.24 (0.16–0.41)	0.22 (0.15–0.31)	0.50 (0.32–0.73)	<0.0001
NLR, median (Q1–Q3)	2.37 (1.25–4.68)	1.82 (1.15–3.19)	6.38 (4.39–9.06)	<0.0001
PLR, median (Q1–Q3)	106.49 (79.95–160.84)	97.01 (74.88–134.3)	206.77 (128.17–307.96)	<0.0001
SII, median (Q1–Q3)	528.57 (302.95–1089.97)	433.84 (265.83–744.45)	1635.22 (1039.23–2795.66)	<0.0001
SIRI, median (Q1–Q3)	1.06 (0.62–2.80)	0.92 (0.56–1.74)	4.31 (2.69–5.66)	<0.0001
AISI, median (Q1–Q3)	265.39 (136.85–666.86)	209.2 (125.01–419.96)	1095.2 (593.47–1721.68)	<0.0001
**Outcomes**				
Mortality, no. (%)	38 (10.30%)	18 (6.12%)	20 (26.67%)	<0.0001 (5.57; 2.77–11.22)
Length of hospital stay, mean ± SD	3.93 ± 0.85	3.93 ± 0.79	4.07 ± 1.06	0.054

AH = arterial hypertension; IHD = ischemic heart disease; AF = atrial fibrillation; CHF = chronic heart failure; MI = myocardial infarction; DM = diabetes mellitus; CKD = chronic kidney disease; PAD = peripheral arterial disease; ICA = internal carotid artery; PCSK9i = proprotein convertase subtilisin/kexin type-9 inhibitors; GFR = glomerular filtration rate; PLT = total platelet count; BUN = blood urea nitrogen; MLR = monocyte-to-lymphocyte ratio; NLR = neutrophil-to-lymphocyte ratio; PLR = platelet-to-lymphocyte ratio; SII = systemic inflammatory index; SIRI = systemic inflammation response index; AISI = aggregate index of systemic inflammation.

**Table 2:** The AUC of the ROC curve, 95% confidence interval, sensitivity, and specificity of the preoperative inflammatory markers.

Variables	Cut-Off	AUC	Std. Error	95% CI	Sensitivity	Specificity	*p*-Value
Restenosis							
MLR	0.30	0.822	0.028	0.767–0.878	80%	74.8%	<0.0001
NLR	3.47	0.890	0.019	0.854–0.927	90.7%	77.9%	<0.0001
PLR	143.05	0.825	0.028	0.771–0.879	72%	79.6%	<0.0001
SII	881.55	0.880	0.021	0.839–0.921	81.3%	80.6%	<0.0001
SIRI	2.01	0.861	0.024	0.814–0.908	81.3%	79.3%	<0.0001
AISI	465.42	0.857	0.026	0.806–0.908	82.7%	77.9%	<0.0001
**Mortality**							
MLR	0.31	0.765	0.044	0.679–0.850	73.7%	70.1%	<0.0001
NLR	4.41	0.885	0.034	0.818–0.953	89.5%	81.6%	<0.0001
PLR	155.07	0.794	0.044	0.708–0.880	78.9%	77.6%	<0.0001
SII	921.47	0.844	0.038	0.769–0.919	86.8%	76.4%	<0.0001
SIRI	2.17	0.819	0.037	0.747–0.891	78.9%	73.1%	<0.0001
AISI	504.97	0.784	0.041	0.704–0.864	76.3%	72.2%	<0.0001

AUC = area under curve; Std = standard; CI = confidence interval; MLR = monocyte-to-lymphocyte ratio; NLR = neutrophil-to-lymphocyte ratio; PLR = platelet-to-lymphocyte ratio; SII = systemic inflammatory index; SIRI = systemic inflammation response index; AISI = aggregate index of systemic inflammation.

**Table 3:** Univariate analysis of inflammatory biomarkers and restenosis risk and mortality.

	Restenosis	Mortality
Low-MLR vs. high-MLR	15/225 (6.67%) vs. 60/144 (41.67%) *p* < 0.0001	10/237 (4.22%) vs. 28/132 (21.21%) *p* < 0.0001
Low-NLR vs. high-NLR	7/236 (2.97%) vs. 68/133 (51.13%) *p* < 0.0001	4/274 (1.46%) vs. 34/95 (57.36%) *p* < 0.0001
Low-PLR vs. high-PLR	21/255 (8.24%) vs. 54/114 (47.37%) *p* < 0.0001	8/265 (3.02%) vs. 30/104 (28.85%) *p* < 0.0001
Low-SII vs. high-SII	14/251 (5.58%) vs. 61/118 (51.69%) *p* < 0.0001	5/258 (1.94%) vs. 33/111 (29.73%) *p* < 0.0001
Low-SIRI vs. high-SIRI	14/247 (5.67%) vs. 61/122 (50%) *p* < 0.0001	8/248 (3.23%) vs. 30/121 (24.79%) *p* < 0.0001
Low-AISI vs. high-AISI	13/242 (5.37%) vs. 62/127 (48.82%) *p* < 0.0001	9/247 (3.64%) vs. 29/122 (23.77%) *p* < 0.0001

MLR = monocyte-to-lymphocyte ratio; NLR = neutrophil-to-lymphocyte ratio; PLR = platelet-to-lymphocyte ratio; SII = systemic inflammatory index; SIRI = systemic inflammation response index; AISI = aggregate index of systemic inflammation.

**Table 4:** Multivariate analysis for predictors of restenosis and mortality at 12 months following CEA.

	Restenosis			Mortality		
	OR	95% CI	*p*-Value	OR	95% CI	*p*-Value
Age > 70	3.26	1.79–5.93	<0.001	5.44	1.59–18.60	0.007
AH	2.30	1.26–4.19	0.006	2.13	0.77–5.85	0.14
IHD	1.42	0.95–2.10	0.08	1.11	0.71–1.73	0.64
AF	1.74	1.06–2.87	0.02	1.26	0.85–1.87	0.24
MI	3.16	1.83–5.44	<0.001	2.83	1.37–5.85	0.005
Tobacco	2.55	1.46–4.46	<0.001	2.31	0.97–5.50	0.058
Obesity	1.90	1.10–3.28	0.02	1.94	0.93–4.05	0.07
Dyslipidemia	5.27	3.07–9.02	<0.001	2.80	1.37–5.71	0.005
Unstable Plaques	2.83	1.67–4.78	<0.001	2.40	1.02–5.63	0.04
Thrombotic Plaques	2.77	1.57–4.90	<0.001	1.73	0.69–4.35	0.24
Antiplatelet	0.46	0.27–0.78	0.004	1.07	0.47–2.46	0.85
Statins	0.59	0.35–0.78	0.04	0.75	0.32–1.72	0.50
Ezetimibe	0.45	0.21–0.95	0.03	0.74	0.24–2.24	0.74
high-MLR	10.37	5.38–18.58	<0.001	6.11	2.86–13.04	<0.001
high-NLR	34.22	14.99–78.12	<0.001	37.62	12.87–109.97	<0.001
high-PLR	12.02	6.62–17.88	<0.001	16.06	5.78–44.61	<0.001
high-SII	18.11	9.46–34.66	<0.001	31.70	10.37–96.84	<0.001
high-SIRI	16.64	8.72–31.74	<0.001	9.89	4.37–22.37	<0.001
high-AISI	16.80	8.69–32.45	<0.001	8.24	3.76–18.08	<0.001

AH = arterial hypertension; IHD = ischemic heart disease; AF = atrial fibrillation; MI = myocardial infarction; MLR = monocyte-to-lymphocyte ratio; NLR = neutrophil-to-lymphocyte ratio; PLR = platelet-to-lymphocyte ratio; SII = systemic inflammatory index; SIRI = systemic inflammation response index; AISI = aggregate index of systemic inflammation.

## Data Availability

Not applicable.
